# Delayed hypersensitivity to nanosecond pulsed electric field in electroporated cells

**DOI:** 10.1038/s41598-017-10825-w

**Published:** 2017-09-08

**Authors:** Sarah D. Jensen, Vera A. Khorokhorina, Claudia Muratori, Andrei G. Pakhomov, Olga N. Pakhomova

**Affiliations:** 1 Frank Reidy Research Center for Bioelectrics, Old Dominion University, Norfolk, VA USA; 2 A. Tsyb Medical Radiological Research Center, Obninsk, Kaluga region Russia

## Abstract

We demonstrate that conditioning of mammalian cells by electroporation with nanosecond pulsed electric field (nsPEF) facilitates their response to the next nsPEF treatment. The experiments were designed to unambiguously separate the electroporation-induced sensitization and desensitization effects. Electroporation was achieved by bursts of 300-ns, 9 kV/cm pulses (50 Hz, n = 3–100) and quantified by propidium dye uptake within 11 min after the nsPEF exposure. We observed either sensitization to nsPEF or no change (when the conditioning was either too weak or too intense, or when the wait time after conditioning was too short). Within studied limits, conditioning never caused desensitization. With settings optimal for sensitization, the second nsPEF treatment became 2.5 times (25 °C) or even 6 times (37 °C) more effective than the same nsPEF treatment delivered without conditioning. The minimum wait time required for sensitization development was 30 s, with still longer delays increasing the effect. We show that the delayed hypersensitivity was not mediated by either cell swelling or oxidative effect of the conditioning treatment; biological mechanisms underlying the delayed electrosensitization remain to be elucidated. Optimizing nsPEF delivery protocols to induce sensitization can reduce the dose and adverse side effects of diverse medical treatments which require multiple pulse applications.

## Introduction

Breaching the cell membrane barrier function by applying high-voltage electric pulses, known as electroporation or electropermeabilization, has been central to a variety of established and emerging treatments in biophysical research, medicine, and biotechnology^1–5^. Optimization of electroporation protocols, including proper choice of pulse duration, amplitude, and repetition rate is essential to achieve the desired outcome (e.g., gene delivery or tumor ablation) with minimal side effects (such as pain and healthy tissue damage).

In most applications, the desired effect of electroporation can only be accomplished by delivering multiple pulses. In that case, the first pulse(s) already modifies (conditions) the target and changes its susceptibility to subsequent pulses.

**Figure 1:**
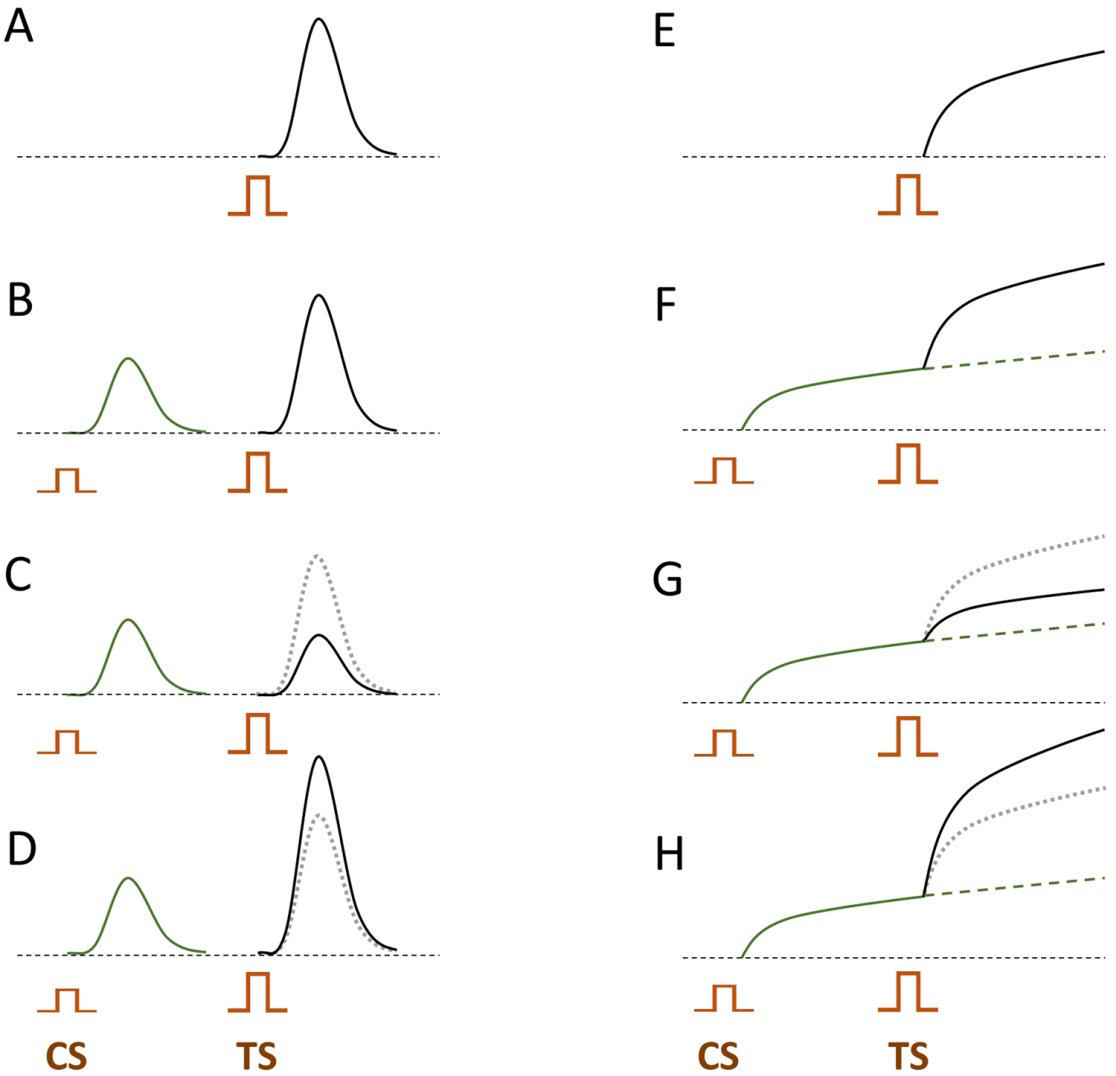
Different effects of conditioning in case of transient effects (**A**–**D**) and long-lasting cumulative effects (**E**–**H**). The top row cartoons (**A** and **E**) show the hypothetical effects of a stimulus applied without conditioning. In other cartoons, the test stimulus (TS) is preceded by a conditioning stimulus (CS). The CS is shown smaller than TS, but it could likewise be made larger or the same as TS. The measured endpoint is the amplitude of the response to TS when it is applied alone or preceded by CS. For easier comparison, the response to TS alone is copied to other panels and shown by a dotted line. Dashed line in panels (F–H) shows the expected development of the CS effect when no TS were applied. In these examples, conditioning had no effect (**B**,**F**); caused desensitization (**C**,**G**); or caused sensitization (**D**,**H**).

Different outcomes of conditioning are explained in the cartoon (Fig. 1). When a test stimulus (TS) is preceded by a conditioning stimulus (CS), the response to TS can stay unchanged, decrease, or increase (Fig. 1B–D). The latter two types of conditioning are called desensitization and sensitization, respectively, and they are distinguished simply by comparing TS responses with and without conditioning.

This approach becomes less straightforward when effects develop over an extended period of time (Fig. 1E–H). In this case, one also needs to know what would be the amplitude of CS effect when no TS is applied (dashed lines in Fig. 1F–H), which usually requires a separate experiment.

The effects of CS and TS may be difficult or impossible to separate when multiple pulses are applied and only their cumulative effect can be measured, such as in cell survival studies. This hindrance may result in heated discussions and diametrically opposite interpretations of the same experimental findings^6–11^, with far-reaching but different implications for science and practical applications.

Specifically, the established mechanism of electroporation is the membrane disruption when the transmembrane potential (TMP) reaches a certain critical level (by different estimates, 0.2–1 V). When an external electric field is applied, the TMP build-up occurs by the accumulation of charges at the membrane interface, known as Maxwell-Wagner ionic polarization. Compared to a naïve membrane, an electroporated membrane is “leaky”; hence the TMP build-up becomes less efficient or impossible. Therefore, repeated electroporating pulses become less and less efficient ( = desensitization), because the transmembrane leak current impedes the TMP build-up. This simple mechanism has been explored by in silico models^9, 12, 13^, and the reduction of desensitization was proposed to explain why lowering the pulse repetition rate (PRR) increases the cumulative effect^9, 10^. Decreasing PRR allows more time for cell membrane resealing between pulses, hence the leak currents become smaller and the TMP build-up efficiency approaches that in naïve cells. Then, the maximum effect of multiples pulses is the additive effect, which is achieved when the pulses are spaced far apart and desensitization is reduced to zero.

However, we found that a high PRR will not reduce the final effect if the entire treatment is split in two fractions with a sufficient wait time between them^8^. The wait time between the two fractions was the key parameter required to achieve greater effect, whereas the PRR itself was not that essential. This phenomenon could not be explained simply by membrane leakage and resealing, leading to a new concept of delayed electrosensitization (DES), caused by an unknown biological mechanism. The DES concept gained further support by demonstrating the supra-additive effect of two sequential treatments^6, 14^. Split dose protocols have shown superior efficiency for drug and dye uptake in cell suspensions^6–8, 11^, 2D and 3D cell cultures^8, 15^, and for tumor ablation *in vivo* ^16^. DES was induced in diverse cell types by nsPEF^8, 15^ as well as by 100-µs electric pulses^6, 7, 11^. The DES concept was also indirectly supported by the observation that repeated electroporation pulses each caused identical dye uptake without signs of desensitization, even though the 1-s interpulse interval tested in this study was far too short for membrane resealing^17^. This experimental result, however, contrasted predictions by a computational method based on a meshed transport network^13^.

When comparing the cumulative effect of multiple pulses, it is easy to confuse DES with the reduction of desensitization, as both would lead to a higher effect at lower PRR. Two recent studies that combined experiments and modeling argued in favor of the reduction of desensitization, but did not test this mechanism with a split-dose protocol^9, 10^. The present study was aimed at resolving the confusion between the two mechanisms, by utilizing the approach illustrated in Fig. 1E–H, which allows for only one unambiguous interpretation. We compared the effect of the same TS (which was a brief burst of pulses) when it was or was not preceded by a CS (another burst of pulses). The electroporation effect was quantified by the cell uptake of propidium (PR) dye which does not penetrate the intact cell membrane. The protocol was designed to accurately measure the PR uptake from CS (PR_CS_) and from TS in naïve and conditioned samples (PR_TS0_ and PR_TS_). Below we demonstrate that conditioning evokes sensitization which can increase the electroporation effect of 300-ns pulses as much as 6-fold. We also quantified DES for diverse conditions and tested for its underlying mechanisms.

## Methods

### Cell culture and sample preparation

**Figure 2:**
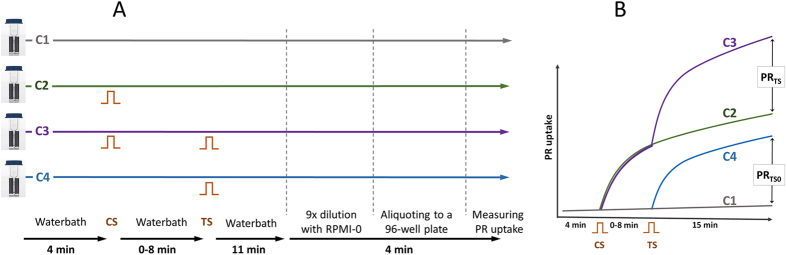
The experiment design and timeline (**A**) and measured endpoints (**B**). A: After aliquoting cell suspension into four electroporation cuvettes (C1-C4) they were placed in a waterbath kept at either 25 or 37 °C. In 4 min, C2 and C3 were subjected to conditioning stimulation (CS) and returned to the waterbath. After a wait time, which was varied from 0 to 8 min, C3 and C4 were subjected to test stimulation (TS) and returned to the waterbath. In 11 min, all suspensions were diluted and processed for measuring propidium (PR) emission in 15 min. (**B**) The expected time course of PR uptake in C1-C4. PR uptake in conditioned and naïve samples (PR_TS_ and PR_TS0_, respectively) was measured as the emission difference between C3 and C2, and between C4 and C1.

U-937 cells (human monocyte lymphoma, ATCC, Manassas, VA) were cultured at 37 °C, 5% CO_2_ in RPMI-1640 medium (Sigma-Aldrich, St. Louis, MO) supplemented with 10% fetal bovine serum (FBS; Atlanta Biologicals, Norcross, GA), 100 units/ml penicillin and 100 µg/ml streptomycin (Mediatech Cellgro, Herdon, VA). Cells were collected in the mid-log phase by spinning for 5 min at 200 G and resuspended to 4 × 10^6^ cells/ml in RPMI-1640 without phenol red, FBS, or antibiotics (this medium is referred below as “RPMI-0”). Cell suspension was placed into a 37 °C, 5% CO_2_ incubator for 15 min. Then, propidium iodide (Sigma-Aldrich) was added to the suspension to 50 µg/ml, and cells were dispensed into four 1-mm gap electroporation cuvettes (BioSmith, San Diego, CA) at 100 µl per cuvette. Such set of four cuvettes constituted one experiment and was prepared for each individual datapoint, without exceptions. The four cuvettes (C1-C4) were subjected to: (C1) sham exposures (same manipulations, but no electric pulses delivered), (C2) CS pulses, (C3) CS followed by TS after a wait time delay, and (C4) TS pulses (Fig. 2A).

### Pulsed electric field (PEF) exposure

Nanosecond (ns) pulses were produced by an AVTECH AVOZ-D2-B-ODA generator (AVTECH Electrosystems, Canada). Pulses of 300 ns duration and 900 V amplitude were delivered with a frequency of 50 Hz to cuvettes via a 50- to 10-Ohm transition module (AVOZ-D2-T, AVTECH Electrosystems) modified into a cuvette holder. The electric field produced in samples was 9 kV/cm, calculated as a voltage-over-distance ratio. To produce pulse trains of exact duration and repetition rate, the AVTECH generator was triggered externally from an S8800 Grass stimulator (Grass Instrument Co., Quincy, MA). The nsPEF amplitude and shape were monitored using a 500 MHz, 5 GS/s TDS 3052B oscilloscope (Tektronix, Wilsonville, OR).

### Experiment timeline and PR uptake measurements

The experiment timeline and procedures are detailed in Fig. 2A. Four cuvettes containing the same cell suspensions were prepared as described above and placed in a waterbath for 4 min. In different sets of experiments, the waterbath was set to either 25 or 37 °C. Two cuvettes, C2 and C3, were exposed to CS and returned to the waterbath. After a wait time, which was varied in different experiments from 0 to 8 min, C3 and C4 were subjected to TS and returned to the waterbath for additional 11 min. Next, cuvettes were taken out of the bath and each sample was diluted with 800 µl of RPMI-0 and mixed gently. Each sample was aliquoted in three wells of a black-sided, clear bottom 96-well plate 96-well, at 200 µl per well. At 15 min after TS, PR emission was measured on a Synergy 2 microplate reader (BioTEK, Winooski, VT), with 530 nm excitation/590 nm emission settings. The data from triplicate wells were averaged and regarded as a single datapoint. Propidium uptake from TS treatment of naïve samples (PR_TS0_) was measured as the difference of fluorescence readings in cuvettes C4 and C1; propidium uptake from TS treatment of conditioned samples (PR_TS_) was measured as the difference of fluorescence readings in cuvettes C3 and C2 (Fig. 2B). The conditioning index (CI) was calculated as a ratio PR_TS_/PR_TS0_ × 100%. By definition, CI < 100% was regarded as desensitization, and CI > 100 as sensitization.

In some sets of the experiments, an additional cuvette (C5) was prepared in the same manner and exposed to CS and TS with no delay between them.

**Figure 3:**
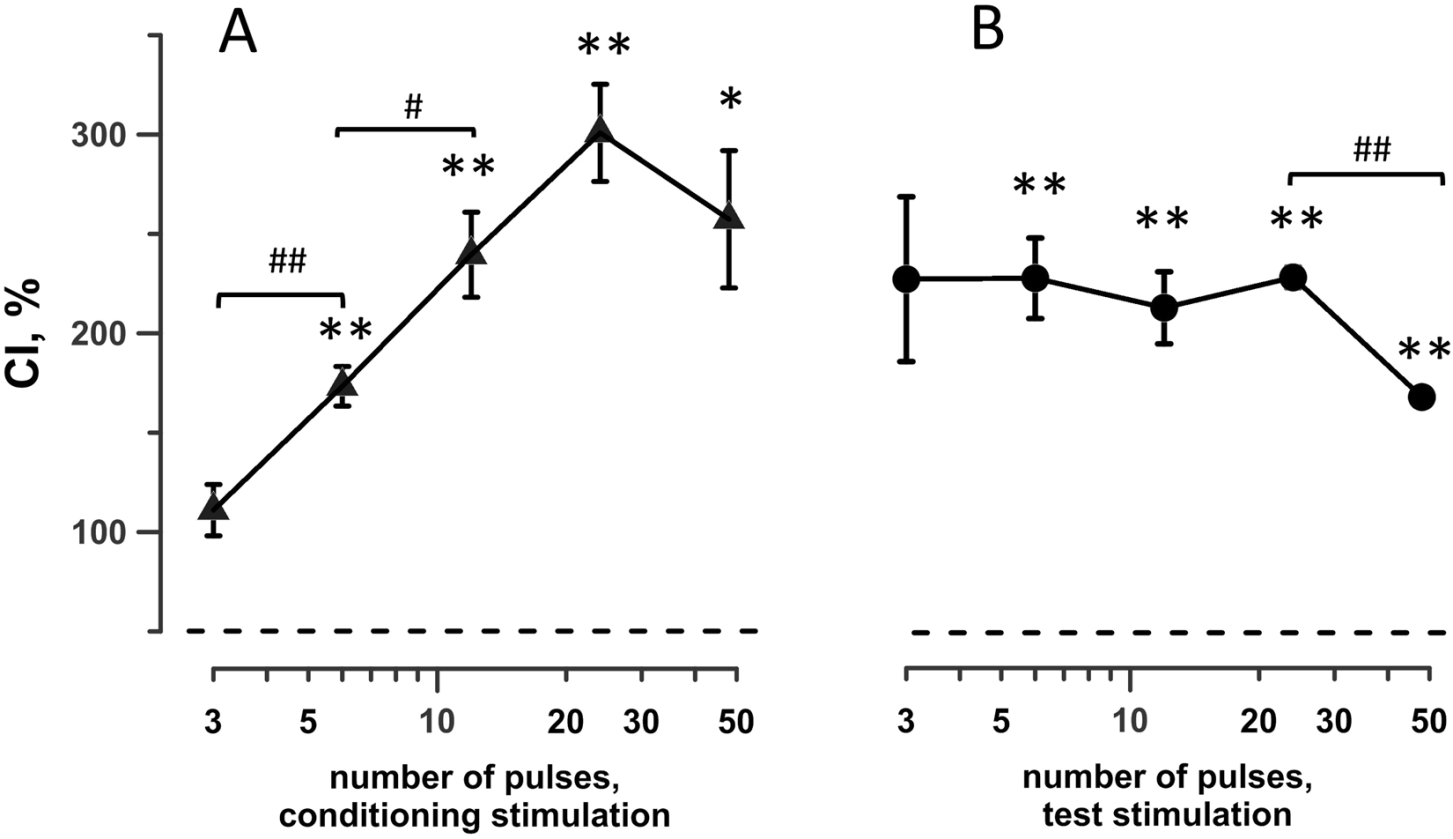
The effect of pulse number in the conditioning stimulation train (**A**) or in the test stimulation train (**B**) on the conditioning index (CI, %). The interval between CS and TS was 2 min and the temperature was 25 °C. 300-ns wide, 9 kV/cm pulses were delivered at 50 Hz. The test train was always 12 pulses (**A**), or the conditioning train was always 12 pulses (**B**). Mean ± s.e.m., n = 4. CI = 100% corresponds to no effect of conditioning and CI > 100% signifies sensitization (*p < 0.05, **p < 0.01, one-sample t-test for the difference from 100%). ^#^p < 0.05, ^##^p < 0.01 for the difference between the groups using two-tailed t-test.

**Figure 4:**
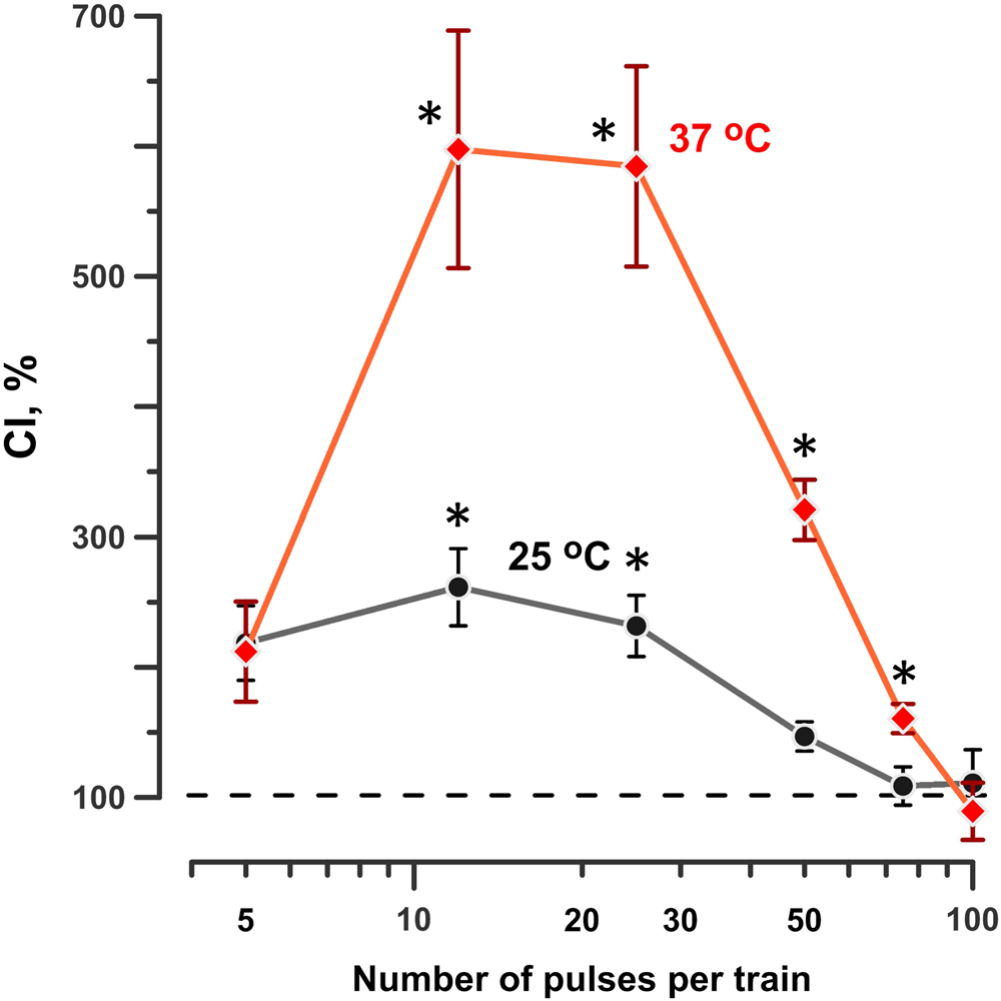
The effect of the pulse number per train on the conditioning index (CI, %). The number of pulses was varied concurrently in both the conditioning and the test trains. The wait interval between the trains was 2 min; the incubation temperature was set to either 25 °C or 37 °C (see legends). Mean ± s.e.m., n = 3. *p < 0.05, one-sample t-test for the difference from 100%. See text and Fig. 3 for more details.

**Figure 5:**
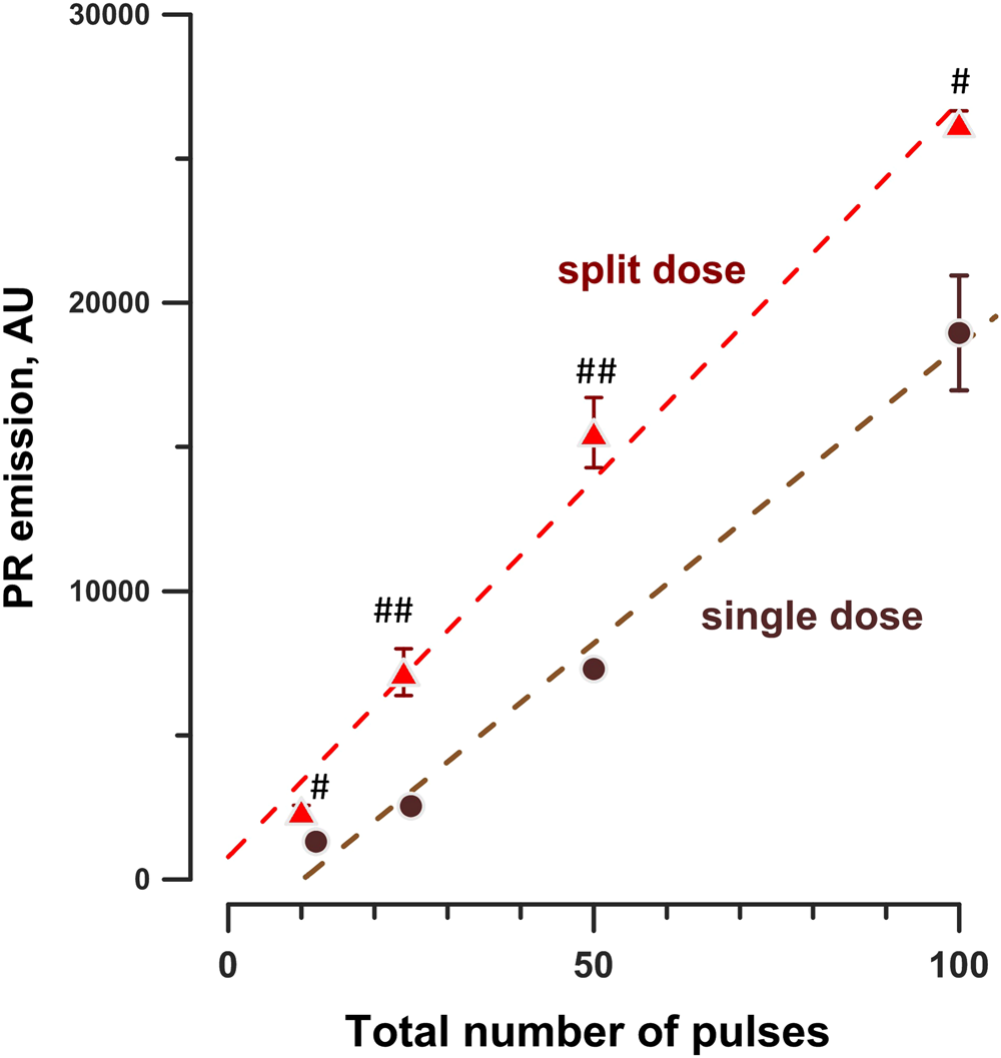
Split dose nsPEF delivery is more efficient for membrane permeabilization than a single dose. Propidium (PR) emission, as measured in 17 min after the first nsPEF treatment, is plotted against the total number of pulses. For the split dose treatment, half of the pulses were delivered by each train; the interval between the trains was 2 min. The incubation temperature was 37 °C. Mean ± s.e.m., n = 3. ^#^p < 0.05, ^##^p < 0.01 for the difference between the two conditions using two-tailed t-test. Error bars may be not visible when they are smaller than the central symbol. See text and Figs 3 and 4 for more details.

**Figure 6:**
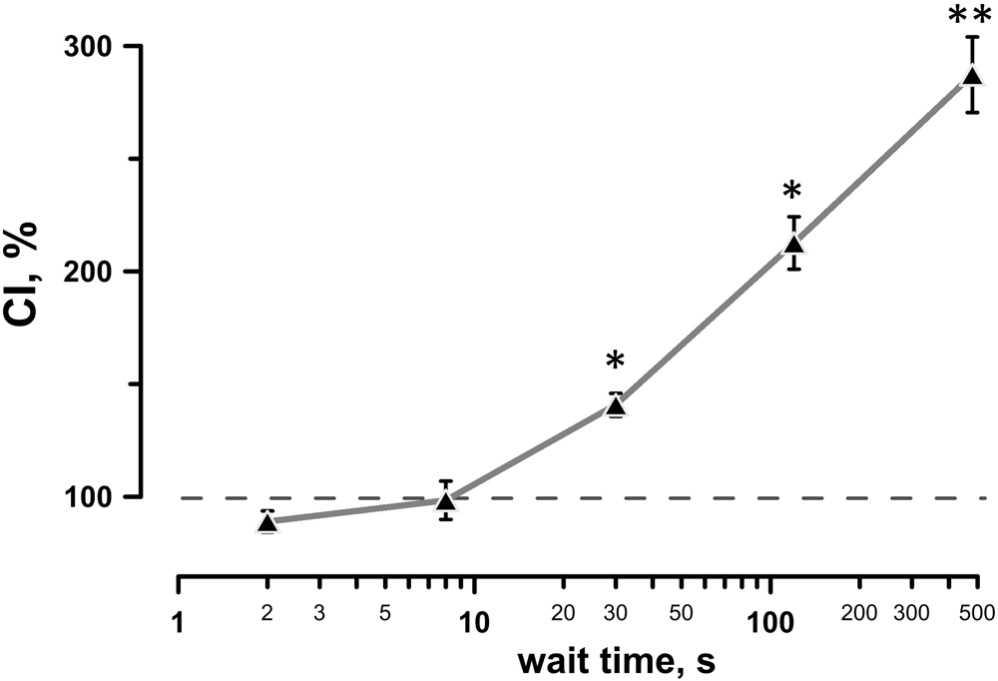
Increasing the wait time after the conditioning nsPEF exposures increases sensitization to the second nsPEF exposure. The conditioning index (CI, %) was plotted against the wait time interval, s. Both the conditioning and test trains consisted of 12 pulses (300-ns wide, 9 kV/cm, 50 Hz). The incubation temperature was 25 °C. Mean ± s.e.m., n = 3. *p < 0.05, **p < 0.01, one-sample t-test for the difference from 100%.

**Figure 7:**
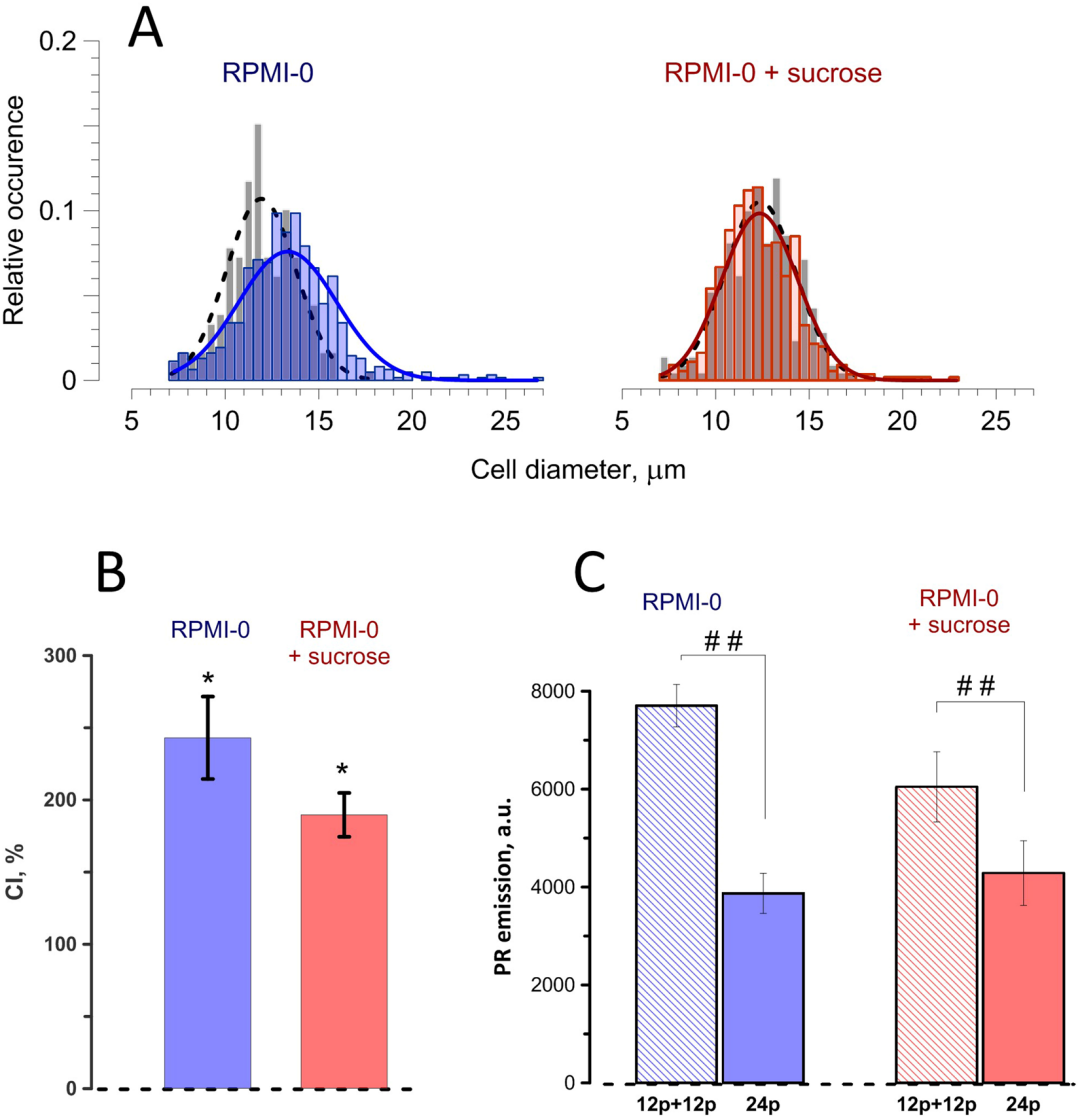
Electrosensitization is not mediated by cell swelling. (**A**) Histograms of cell diameter distribution at 2 min after a sham exposure (solid gray bars) or permeabilization with 12 pulses (300-ns, 9 kV/cm, 50 Hz; semi-transparent colored bars). Dashed and solid lines are the normal (Gaussian) distribution best fits for sham and nsPEF exposure data, respectively. Note diameter increase in RPMI-0 medium (left panel) and its blockage in the presence of sucrose (right). (**B**) Cells in both media displayed strong sensitization, as manifested by the CI of about 200%; mean +/− s.e., n = 4, *p < 0.05, one-sample t-test for the difference from 100%. The interval between conditioning and test treatments (each of 12 nsPEF) was 2 min, at 25 °C. (**C**) Split-dose nsPEF delivery protocol (12 pulses + 12 pulses in 2 min) was more efficient than a single dose of 24 pulses in both types of media. Mean ± s.e.m., n = 5. ^##^p < 0.01 with a two-tailed t-test.

**Figure 8:**
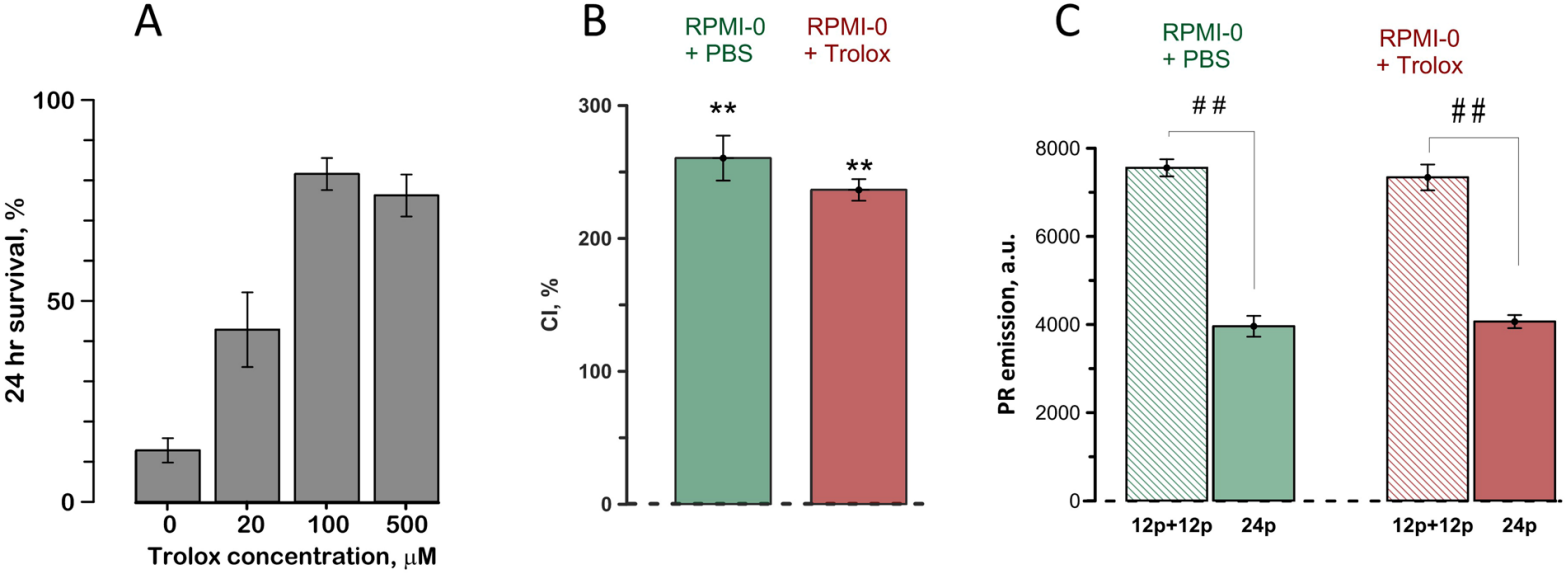
Electrosensitization is not prevented by Trolox. (**A**) 100 µM of Trolox was most efficient to prevent cell loss from incubation with 100 µM cumene hydroperoxide for 2 hours. Cell survival was measured 24 hr later and expressed in % to vehicle-treated parallel controls. Mean +/− s.e., n = 4. See text for more details. (**B**) Trolox did not prevent sensitization, as manifested by the CI of over 200%; mean +/− s.e., n = 3, **p < 0.01, one-sample t-test for the difference from 100%. The interval between conditioning and test treatments (each of 12 nsPEF, 300-ns, 9 kV/cm, 50 Hz) was 2 min, at 25 °C. (**C**) Trolox did not change the higher efficiency of a split-dose nsPEF delivery (12 pulses + 12 pulses in 2 min) compared to a single dose of 24 pulses. Mean ± s.e.m., n = 4, ^##^p < 0.01 with a two-tailed t-test.

Each individual datapoint on graphs in Figs 3–8 below is the mean +/− s.e.m. of 3–4 independent experiments such as described above. In each set of experiments, different treatment conditions were alternated in a random manner.

### Cell size measurements

Cell diameters were measured using Cellometer Vision (Nexcelom Biosciencea, Lawrence, MA). In 2 min after CS treatment, the cells were transferred into a counting chamber and imaged with transillumination light. The images were processed using Cellometer software. Diameters of approximately 300 cells were measured per experiment. In these experiments, the starting cell density was 0.4 × 10^6^ cell/ml and the samples were diluted twofold before measurements.

### Inhibition of cell swelling

Post-exposure cell swelling was blocked with sucrose, which does not enter nanoporated cells and thereby efficiently counters the colloid-osmotic imbalance and swelling after nsPEF^18–20^. RPMI-0 was mixed 3:1 with an isosmotic solution of sucrose in water (280 mOsm/kg), so that the fractional osmolality due to sucrose in the mixture was 90 mOsm/kg^19, 20^. Cells were placed in this mixture prior to nsPEF treatments, and the waterbath was set to 25 °C.

### Blockage of lipid oxidation

Membrane lipid oxidation was blocked with Trolox (6-hydroxy-2,5,7,8-tetramethylchromane-2-carboxylic acid; Sigma-Aldrich). To find the effective yet non-toxic concentration of Trolox, it was tested for protection from a benchmark oxidizing agent, cumene hydroperoxide (CH; part of The Image-iT Lipid Peroxidation Kit, ThermoFisher Scientific, Waltham, MA). On the day of experiments, CH stock was prepared by adding 1 µl of 5.4 M CH solution in DMSO to 54 µl of 100% ethanol. Vehicle stock was prepared with 1 µl DMSO and 54 µl of 100% ethanol. Trolox stock was prepared as a 10 mM solution in PBS, pH adjusted to pH 7.5 with NaOH, and sterile filtered.

Cells were prepared at 5 × 10^6^ cells/ml in the growth medium and incubated with different Trolox concentrations (0 to 500 µM) for 1 hour at 37 °C prior to the addition of CH 100 µM for 2 hours. Equivalent volumes of vehicle were added as parallel controls. Next, cells were spun and rinsed twice with fresh growth medium and placed in the incubator for 24 hours. Cell survival was determined with a Presto Blue metabolic assay (ThermoFisher Scientific).

For experiments with nsPEF, cells were prepared at 4 × 10^6^ cells/ml in RPMI-0 with 100:1 addition of either the Trolox stock solution or PBS as a vehicle control. Samples were placed in the incubator for 1 hour and then subjected to nsPEF treatments as described above in section 2.3 and in Fig. 2. The waterbath was set to 25 °C.

### Statistical analysis

The statistical significance of deviations of CI values from 100% was determined with a one sample *t*-test. The mean data values for different groups were compared with an unpaired two-tailed *t*-test. Data are presented in graphs as mean values +/− s.e.m. for *n* independent experiments; p < 0.05 was considered statistically significant. The graphs were generated with Grapher 11 (Golden Software, Golden, CO).

### Data availability

All data generated or analysed during this study are available from the corresponding author on reasonable request.

## Results

### The effect of pulse number

In the first set of experiments, cells were conditioned by different numbers of pulses (from 3 to 50), whereas the TS treatment was kept constant at 12 pulses, The wait time between CS and TS was always 2 min and the waterbath was set to 25 °C. Except for 3 pulses, conditioning resulted in 2–3-fold increase (p < 0.01) of the response to TS (Fig. 3A). The sensitization became significantly stronger as the number of conditioning pulses increased from 6 to 24. Further increase of the CS to 48 pulses did not enhance sensitization any further, possibly indicating that the saturation has been reached and/or that damages from a too intense CS hinder the sensitization effect.

The next set of experiments was the same, except for now the conditioning dose was kept constant at 12 pulses, whereas the TS was varied from 3 to 48 pulses (Fig. 3B). The conditioning index did not depend on the number of pulses in the TS and stayed constant at 200–250% for all treatments except for the possibly saturating dose of 48 pulses.

Within studied limits, these experiments established that (1) sufficiently intense conditioning always caused sensitization and never desensitization, (2) the sensitization effect increased with more intense conditioning, up to a certain limit, and (3) the facilitation of the response of already sensitized cells did not depend on the intensity of the test stimulation, also up to a certain limit.

### Effect of the total pulse number and temperature

Now, the number of pulses was changed concurrently in both CS and TS, from 5 to 100 in each of the treatments. At 25 °C, strong sensitization (CI > 200%) was achieved with 5, 12, and 25 pulses per treatment (Fig. 4). At 50 pulses, the effect dropped to 140% and disappeared for still more intense treatments, consistent with the saturation or damage mechanism suggested above.

Increasing the incubation temperature to 37 °C has enhanced sensitization to as much as 600%, which was at least 2-fold more than we could achieve at the lower temperature. The dependence of the CI on the number of pulses was similar to 25 °C data (Fig. 4). Stronger sensitization at the physiological temperature is important for practical applications of nsPEF *in vivo*, and supports the notion that the electroporation-induced sensitization is a biological rather than simply a physicochemical effect^8^.

### The importance of the wait time between the conditioning and test treatments

One of key findings from our earlier studies^7, 8^ was that splitting an exposure into two fractions with a proper wait time between them increases the overall effect. Data from the previous set of experiments prove that this conclusion also holds true at 37 °C and for a broad range of pulse numbers. For this analysis, cuvette 2 (C2) data were compared with cuvette 3 (C3) data from other experiments, in which C3 was treated by a matching total number of pulses. The highest number of pulses delivered to C2 was 100, hence C3 data for more than 100-pulse treatments (75 + 75 and 100 + 100 pulses) were not considered. Since C2 received the exposure as a single dose, and C3 received it as two doses (CS + TS), their comparison emphasizes the impact of the 2-min wait time between the doses. For all datapoints, the effect was stronger in samples subjected to a split-dose treatment (Fig. 5), consistent with the previous reports^7, 8^. Of note, the effect in C3 was stronger even despite the fact that the total time for PR entry after the full dose was 2 min less than in C2 (see ref. 7 for a detailed discussion of this caveat).

The impact of the wait time was explored further in separate experiments, where both CS and TS were kept constant at 12 pulses, and the wait time was varied from 2 to 480 s (Fig. 6). Sensitization was measured as the CI, the same way as described above in the Methods and in Fig. 2. Cuvettes were kept at a room temperature. With 2- and 8-s wait times, no effect of conditioning was observed (CI decrease to below 100% with 2-s wait time could be indicative of desensitization, but the difference from 100% was not statistically significant). A 30-s wait time was already sufficient for the development of sensitization, and still longer intervals increased the effect of conditioning.

### Exploring mechanisms of electrosensitization: role of electroporation-induced cell swelling

The membrane potential induced by an external electric field at the poles of a round-shaped cell is linearly proportional to its diameter^1–3^. Therefore, with all other variables kept the same, larger cells should be more susceptible to electroporation. Cell swelling due to the colloid-osmotic imbalance is a well-established effect of electroporation^18–23^, and it develops gradually on approximately the same time scale as electrosensitization after the CS (Fig. 6). Therefore, it was logical to hypothesize that swelling of cells after the conditioning nsPEF exposure resulted in their delayed hypersensitivity to the test nsPEF.

To test this mechanism, the colloid-osmotic imbalance was countered by adding an electropore-impermeable solute (sucrose) to the medium, as described above in 2.3. Both CS and TS were trains of 12 pulses each, with a 2-min interval. One more cuvette with cell suspension was prepared in addition to 4 cuvettes shown in Fig. 2; this cuvette was exposed to CS and TS without any wait time between them (i.e., it was exposed to a train of 24 pulses). Control experiments using the standard medium (RPMI-0) and experiments using RPMI-0 with sucrose were alternated in a random manner.

Electroporation by 12 pulses in the RPMI-0 medium caused cell swelling, with the average cell diameter increase of 12% by 2 min after the treatment. The presence of sucrose blocked swelling completely (Fig. 7A). However, the inhibition of swelling did not prevent electrosensitization: The CI decreased modestly (from 243 +/− 30% in RPMI-0 to 190+/− 25% in RPMI-0 + sucrose) and sensitization remained statistically significant (p < 0.02, one sample t-test, Fig. 7B).

Likewise, comparing the effect of 24 pulses delivered as a single dose (cuvette 5) to the effect of a split-dose delivery with a 2-min wait time (cuvette 3) revealed a large enhancement of PR uptake after the split-dose treatments in both tested media (p < 0.01, paired t-test; Fig. 7C). Thus we conclude that electrosensitization was not caused by cell swelling after the conditioning nsPEF exposure.

### Exploring mechanisms of electrosensitization: possible oxidative damage to cell membrane by nsPEF

Although cell membrane permeabilization is the established principal mechanism of nsPEF bioeffects, it is not the only mechanism. Cell survival studies suggested that nsPEF effects might be mediated by electrochemical effects, possibly including ROS formation^24, 25^. Later studies demonstrated that nsPEF triggers oxidation both extracellularly (in a process that does not require the presence of cells) and intracellularly, in a biologically-mediated pathway^26, 27^. Separate studies found that oxidatively damaged areas of the cell membrane may be more sensitive to electroporation^28^. When taken together, these findings made it logical to hypothesize that the conditioning treatment causes oxidation of cell membrane lipids, thereby rendering them more vulnerable to the second application of nsPEF.

If this were indeed the mechanism of sensitization, chemical inhibition of membrane oxidation would prevent or attenuate sensitization. Here we tested if such inhibition is achieved with Trolox, a water-soluble analog of vitamin E.

As a positive control, we first established Trolox concentration for an efficient protection against a known chemical oxidant, cumene hydroperoxide. At 100 µM, Trolox rendered maximum protection without reducing cell survival (Fig. 8A), hence this concentration was chosen for sensitization studies. We employed the same experiment protocol as described in the previous section to compare sensitization in the RPMI-0 with Trolox or with PBS as a vehicle. These experiments established that the presence of Trolox did not change the CI (Fig. 8B) and the split dose treatments remained about twice more efficient as single-dose exposures (Fig. 8C). Within studied limits, these experiments did not support the oxidative mechanism of electrosensitization.

## Discussion

We successfully utilized 300-ns, 9 kV/cm electric pulses, arranged into brief 50-Hz trains, to electroporate mammalian cells and change their sensitivity to subsequent electroporation treatments. The new experiment protocol introduced and employed in this study enabled unbiased quantification of the conditioning effect under diverse treatment conditions. We varied the number of pulses per train (and, consequently, the severity of electroporation), the wait time between the trains, and the incubation temperature. The uptake of propidium dye by electropermeabilized cells served as a quantitative benchmark of the electroporation efficiency.

The overarching goal of this study was to distinguish between sensitization and desensitization following a conditioning treatment with nsPEF. Under all conditions tested, we observed either sensitization or no effect (when the conditioning was either too weak or too intense, or when the wait time was not sufficient). Surprisingly, we have not observed desensitization under any of tested conditions, although it has a simple and logical physical rationale, and was predicted by theoretical analyses^9, 10, 13^. One may speculate that physical desensitization due to the increased membrane leakiness after the conditioning electroporation was present, but its impact was dwarfed by the biological effect of sensitization. If this is the case, desensitization can possibly be revealed with very short wait times which do not allow for sensitization to develop, or after the inhibition of sensitization. Earlier work indicated that sensitization can be inhibited in certain media^6^, but no mechanistic connection was made.

We tested two hypothesized mechanisms which could cause sensitization (cell swelling and membrane oxidation), but found no proof for either one. Other mechanisms considered and remaining to be tested include influx of Ca^2+^ and its downstream effects, cytoskeleton disruption, and ATP leakage and depletion^6, 8^. A recent study suggested that electrosensitization could result from a dose-dependent activation of phosphatidylinositol-4,5-bisphosphate signaling^29^, but the time course of this effect was different from the delayed development of sensitization.

An interesting yet unexplored possibility is the connection between electrosensitization and cell membrane repair, which is an active biological process occurring on a similar time scale^30^. It is logical to hypothesize that, while being repaired, the membrane is in an unstable condition and can be easier disrupted. Profound enhancement of sensitization that was observed at 37 °C is also consistent with its possible connection to the activation of repairs. This enhancement is also the most welcome news for cancer and tissue ablation medical applications of nsPEF. Indeed, the 6-fold enhancement of the electroporation effect, which was achieved by conditioning cells at 37 °C, will enable ablation at lower electric fields or with fewer pulses, thereby reducing adverse side effects, such as pain and muscle contractions. Alternatively, sensitization may help ablation of larger areas with fewer repositionings of the electrodes. For all these treatments, sensitization can be engaged by simply using split-dose pulse delivery protocols instead of a single dose. As a matter of fact, split-dose protocols are already used for ablation (e.g., ref. 31) to allow extra time for heat dissipation and to prevent undesirable thermal effects; electrosensitization is an additional reason to choose them.
